# Supplementary material for: Does Coenzyme Q10 Supplementation Improve Testicular Function and Spermatogenesis in Male Mice with Chronic Kidney Disease?
Source: Biology (Basel). 2021 Aug 17;10(8):786. doi: 10.3390/biology10080786 (PMC8389647; doi:10.3390/biology10080786)
Supplement: Supplementary file 1 [file biology-10-00786-s001.zip › biology-1294442-supplementary.pdf]

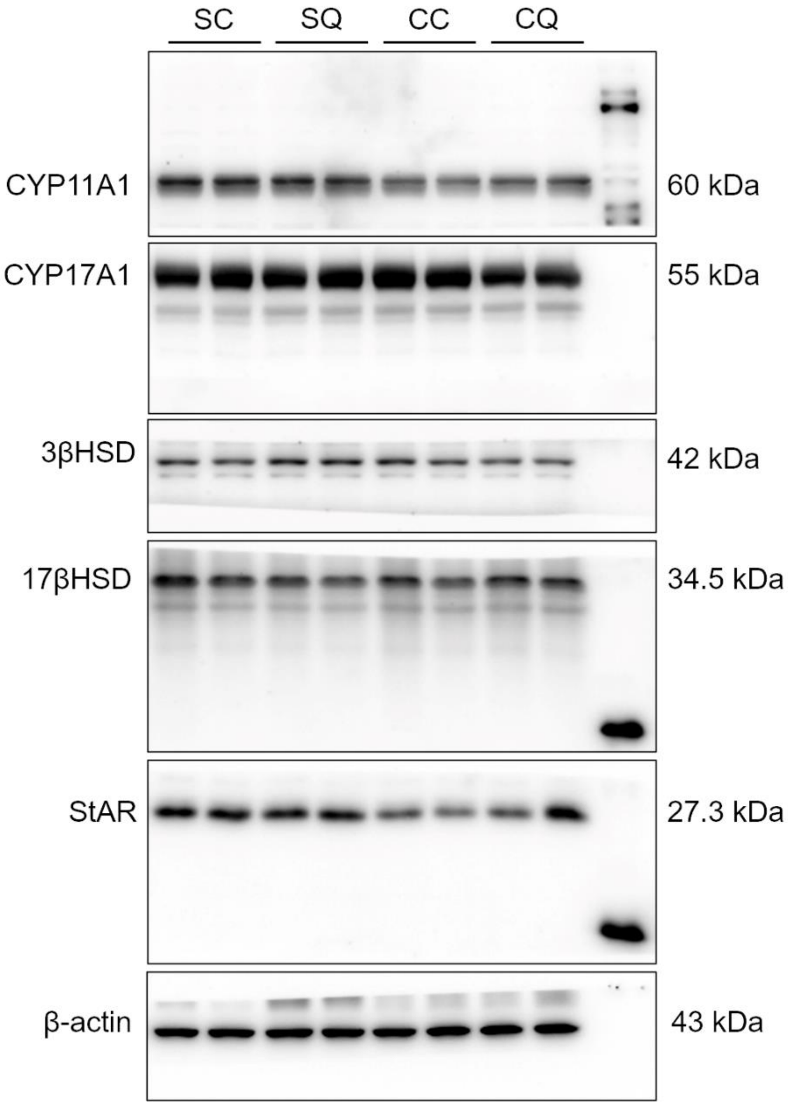

	SC		SQ		CC		CQ	
CYP11A1/β-actin	0.836	0.939	1.027	0.963	0.721	0.597	0.672	0.726
CYP17A1/β-actin	1.139	1.070	0.997	1.064	1.003	0.977	0.849	0.834
3βHSD/β-actin	0.759	0.768	1.097	1.031	0.989	0.801	0.705	0.661
17βHSD/β-actin	0.940	0.837	0.871	0.720	0.936	0.750	0.898	0.837
StAR/β-actin	1.064	1.031	0.941	1.026	0.759	0.608	0.652	0.914

Figure S1. Whole western blot and densitometry readings/intensity ratio of testosterone biosynthesis enzymes in the sham surgery and CKD surgery mice treated with vehicle (corn oil) or CoQ10.

SC: sham surgery mice with vehicle; SQ: sham surgery mice with CoQ10; CC: CKD surgery mice with vehicle; CQ: CKD surgery mice with CoQ10.

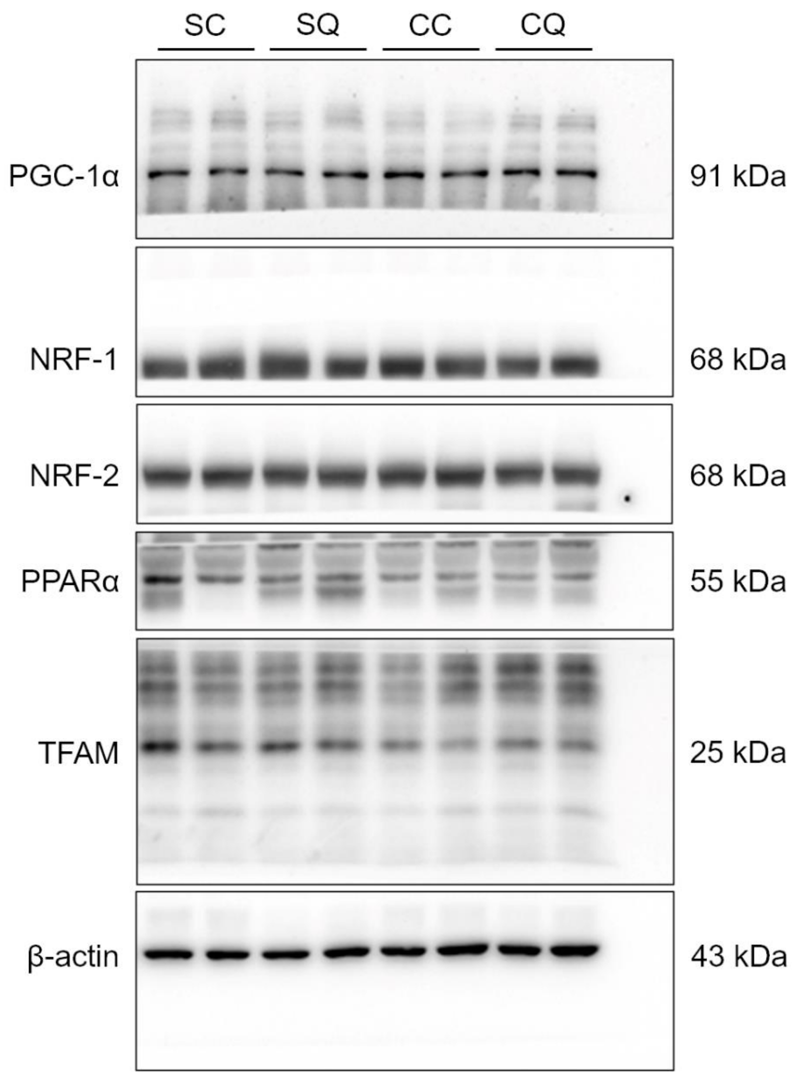

	SC		SQ		CC		CQ	
PGC-1α/β-actin	0.813	0.845	0.789	0.891	0.978	0.718	0.754	0.660
NRF-1/β-actin	0.898	1.241	1.393	0.975	1.180	0.942	0.864	0.869
NRF-2/β-actin	1.128	1.201	1.024	0.963	1.057	1.090	0.939	0.967
PPARα/β-actin	0.761	0.483	0.719	0.837	0.459	0.523	0.415	0.456
TFAM/β-actin	0.723	0.597	0.752	0.698	0.528	0.421	0.535	0.513

Figure S2. Whole western blot and densitometry readings/intensity ratio of testicular mitochondria biogenesis markers in the sham surgery and CKD surgery mice treated with vehicle (corn oil) or CoQ10.

SC: sham surgery mice with vehicle; SQ: sham surgery mice with CoQ10; CC: CKD surgery mice with vehicle; CQ: CKD surgery mice with CoQ10.
